# Three-year follow-up of Interleukin 6 and C-reactive protein in chronic obstructive pulmonary disease

**DOI:** 10.1186/1465-9921-14-24

**Published:** 2013-02-20

**Authors:** Renata Ferrari, Suzana E Tanni, Laura MO Caram, Corina Corrêa, Camila R Corrêa, Irma Godoy

**Affiliations:** 1 Faculdade de Medicina de Botucatu, Univ Estadual Paulista, Unesp, Disciplina de Pneumologia, Botucatu, São Paulo, Brazil

**Keywords:** Inflammation, Biomarkers, Exercise, Chronic Obstructive Pulmonary Disease

## Abstract

**Background:**

Past studies have shown that mean values of Interleukin-6 (IL-6) and C-reactive protein (CRP) do not change significantly in COPD patients over a one-year period. However, longer period follow-up studies are still lacking. Thus, the aim of this study is to evaluate plasma CRP and IL-6 concentration over three years in COPD patients and to test the association between these inflammatory mediators and disease outcome markers.

**Methods:**

A cohort of 77 outpatients with stable COPD was evaluated at baseline, and 53 (mean FEV_1_, 56% predicted) were included in the prospective study. We evaluated Interleukin-6 (IL-6), C-reactive protein (CRP), six-minute walking distance (6MWD), and body mass index (BMI) at baseline and after three years. Plasma concentration of IL-6 was measured by high sensitivity ELISA, and CRP was obtained by high sensitivity particle-enhanced immunonephelometry.

**Results:**

IL-6 increased significantly after 3 years compared to baseline measurements [0.8 (0.5-1.3) vs 2.4 (1.3-4.4) pg/ml; p < 0.001] and was associated with worse 6MWD performance. In the Cox regression, increased IL-6 at baseline was associated with mortality [Hazard Ratio (95% CI) = 2.68 (0.13, 1.84); p = 0.02]. CRP mean values did not change [5 (1.6-7.9) vs 4.7 (1.7-10) pg/L; p = 0.84], although eleven patients (21%) presented with changes >3 mg/L in CRP after 3 years.

**Conclusions:**

The systemic inflammatory process, evaluated by IL-6, seems to be persistent, progressive and associated with mortality and worse physical performance in COPD patients.

**Trial registration:**

No.:NCT00605540

## Introduction

Chronic obstructive pulmonary disease (COPD) affects primarily the lungs; however, it is now recognized as a disease with systemic repercussions, and it is associated with chronic inflammation [1]. In fact, there is increasing evidence that systemic inflammatory mediators such as C-reactive protein (CRP) and interleukin 6 (IL-6) are increased in the peripheral blood of COPD patients [2,3]. Cross-sectional studies show that CRP levels are related to important clinical outcomes, including exercise tolerance [4], health status [5] and the exacerbation of disease [6]. Plasma IL-6 concentration is known as a powerful promoter of CRP production in the liver [7], and it is associated with CRP levels in COPD patients [4,5,8]. IL-6 levels also have been shown to interfere with malnutrition pathophysiology since is increased in low weight COPD patients [9]. A previous study has shown that mean values of CRP remained stable over a 17- month period [10]; in addition, Kolsum et al. [8] have shown that IL-6 did not change over one-year.

Recent study showed that systemic inflammation, when present for at least 1 year, was associated with a higher incidence of exacerbations and worse survival despite similar lung impairment in COPD patients [11]. Furthermore, survival analyses show that the addition of white blood cell counts and the systemic levels of IL-6, CRP, interleukin 8, fibrinogen, chemokine ligand 18, and surfactant protein D improve significantly the ability of clinical variables to predict mortality in COPD patients [12]. In a cohort of 253 COPD patients, the results showed that the highest levels of inflammatory markers was related to the degree of airflow obstruction, functional capacity and health status [13]. The importance of studies to evaluate the inflammatory status in COPD has increased since the launching of new drugs, such as an oralphosphodiesterase 4 (PDE4) inhibitor with systemic effects and appears to offer the potential to target the inflammatory processes underlying COPD [14]. Clinical trials have demonstrated that roflumilast improves lung function and reduces exacerbation frequency in COPD.

In summary, systemic inflammation in COPD is associated with poor outcomes and long-term follow-up studies of inflammatory mediators are needed to better understand the progression of this outcome. We hypothesized that reporting the evolution of CRP and IL-6 will provide information regarding the utility of these mediators in clinical practice and consequently to guide therapeutic interventions in COPD. Thus, the aim of this study was to evaluate plasma CRP and IL-6 concentration over three years in COPD patients and to test the association between these inflammatory mediators and nutritional status, exercise tolerance, disease exacerbations and mortality.

## Materials and methods

### Study population

Seventy-seven patients with mild to very severe COPD attending the Botucatu Medical School in Brazil were evaluated, and 53 participated in the prospective study. Major inclusion criteria included a diagnosis of COPD according to GOLD 2006 and the Brazilian Thoracic Society (BTS) guidelines [1,15]. Exclusion criteria included a primary diagnosis of other respiratory diseases or chronic diseases, recent (<4 months) myocardial infarction, unstable angina or congestive heart failure (New York Heart Association class III or IV). Patients not clinically stable (changes in medication, disease exacerbation, or hospital admissions in the preceding 6 weeks) were also excluded. Patients were evaluated at baseline and were interviewed by telephone every 3 months to identify data associated with exacerbation and/or hospitalizations (see the Additional file 1). The study was approved by the Research Ethics Committee of Botucatu Medical School University Hospital (390/2007), and all patients signed an informed consent.

### Measurements

#### Spirometry and pulse oximeter oxygen saturation (SpO_2)_

Pre and post bronchodilator spirometry was performed (Ferrari KOKO Louisville, CO 80027, USA) according to criteria set by the American Thoracic Society [16]. SpO_2_ was assessed using an Onyx oxymeter (Model 9500 Oximeter; Nonin Medical Inc.; Minneapolis, MN, USA) on room air.

#### Blood sampling and analysis

Fasting peripheral blood was collected (08.00 hours), and plasma was stored at −80°C. IL-6 was assessed in duplicate by high sensitivity commercial kits using enzyme linked immunosorbent assay (ELISA) according to the manufacturer's instructions (BioSource International Inc, Ca, USA) with a lower detection limit of 0.16 pg/mL. CRP was assessed in duplicate by high sensitivity particle enhanced immunonephelometry (Cardio-Phase, Dade Behring Marburg GmbH, Marburg, USA) with a lower detection limit of 0.007 mg/L. All measurements were performed after the final evaluation using kits of the same lot number to avoid measurement bias.

#### Exercise tolerance

The six-minute walk distance (6MWD) was performed according to the guidelines of the American Thoracic Society [17] (see the Additional file 1).

#### Nutritional status, dyspnea perception, multidimensional index, comorbidity index

Body weight and height were measured, and body mass index [BMI = weight in kg/(height in m)^2^ was calculated. Dyspnea was assessed using a translated version of the Modified Medical Research Council (MMRC) scale [18]. BODE index was calculated using the model described by Celli et al. [19]. Comorbidities were quantified by the Charlson index [20].

### Statistical analysis

All analyses were performed using SigmaStat 3.2 (Inc, Chicago, IL, USA) and R statistical software [21]. Multivariate Cox regression analysis was performed to evaluate the predictors of mortality including all the subjects evaluated at baseline, adjusting for age, gender, BODE index, SpO_2_ and comorbidity index. Paired t-test or Wilcoxon test was performed to compare characteristics at baseline and after three years. For baseline characteristics, the unpaired *t*-test or the Mann–Whitney test was used. Spearman correlations were used to determine the relationships between inflammatory mediators. Two multiple linear regression analyses were performed including gender, age, FEV_1_, IL-6 and Charlson index, at baseline, as independent variables and the baseline and final values of 6MWD as the dependent variables. The same model was used with BMI and number of exacerbations as the dependent variables. A p ≤ 0.05 was defined as statistically significant.

## Results

The baseline characteristics of the 77 patients (66% men; FEV_1_ = 57%) were mean age of 64 ± 9 years and smoking exposure of 51 ± 29 pack-years; 24 patients (31%) were active smokers. Sixty-five patients were using long-term bronchodilators, and 17 patients were regularly using inhaled corticosteroids; 16 had been on stable oxygen flow therapy for the last six months. Comparison between patients using or not inhaled corticosteroids at baseline and after three years showed no significant difference for the studied variables. No patients were medicated with theophylline or leukotriene modifiers.

**Figure 1:**
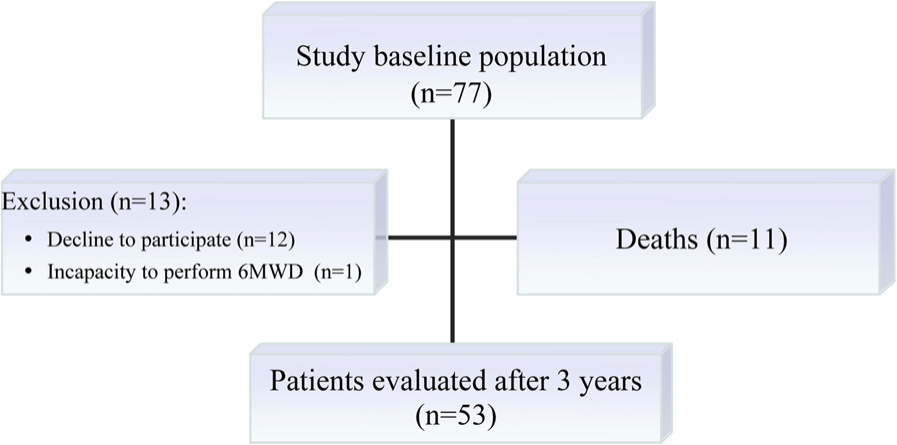
Diagram of patient follow up in three-year period. 6MWD = six-minute walking distance.

Of the 77 patients initially evaluated, 24 were excluded from the final analyses; 11 patients died and 13 dropped out (Figure 1). The causes of deaths were pulmonary complications resulting from COPD in 4 patients, cardiovascular disease in 5 patients, splenic abscess/septic shock in one patient, and colon cancer in one patient. Thus, 53 patients were monitored for three years.

At baseline, the mean age of the 53 patients included in the prospective study (66% men) was 63 ± 9 years with smoking exposure of 48 ± 27 pack-years; 16 patients (30%) were active smokers. Eight patients (15%) were in GOLD stage I, 19 (36%) were in stage II, 11 (20%) were in stage III, and 15 (28%) were in stage IV COPD. There was no difference in the proportion of patients within each disease severity between baseline and after three years (p = 0.99). The comparison of 53 patients’ characteristics between baseline and after three years is shown in Table 1 and has been included in a previous publication with 95 patients [22].

**Table 1:** Characteristics of COPD patients followed for a three-year period

**Variables**	**Initial assessment (n = 53)**	**Final assessment (n = 53)**	**p-value**
FEV_1_ (%)	56.0 ± 21.1	56.0 ± 22.6	0,48
FEV_1_ (l)	1.4 ± 0.7	1.3 ± 0.6	**0.03**
FVC (%)	88.9 ± 20.9	86.3 ± 24.3	0.20
FVC (l)	2.6 ± 0.9	2.5 ± 0.9	**0.01**
FEV_1_/FVC	50.5 ± 12.1	50.2 ± 10.6	0.49
SpO_2_ (%)	93.0 ± 3.1	91.6 ± 4.9	**0.02**
MMRC (score)	1.6 ± 1.0	2.0 ± 1.2	**0.01**
6MWD (m)	444.0 ± 82.8	406.3 ± 102.9	**<0.001**
BMI (kg/m^2^)	25.6 ± 5.5	25.5 ± 5.4	0.38
BODE index	2.4 ± 1.9	2.9 ± 2.5	**0.01**

Comparisons of patient characteristics at baseline and after three years were performed by Paired t-test or Wilcoxon; p ≤ 0.05. Values are presented as mean ± SD. *Definition of abbreviations*: FEV_1_ = forced expiratory volume in the first second (% of predicted); FVC = forced vital capacity (% of predicted); SpO_2_ = pulse oximeter oxygen saturation; MMRC = Modified Medical Research Council; 6MWD = six-minute walking distance; BMI = body mass index.

In summary, the FEV_1_ and FVC values, in liters, decline statistically during the study period. There were significant deteriorations of SpO_2_, MMRC, 6MWD, and BODE index. The Charlson index increased significantly in the period (3.0 ± 1.1 vs. 3.4 ± 1.2, p = 0.02). However, in 14 patients, the increase was attributable only to the change in the patient age. Forty-one patients (77.3%) had at least one exacerbation during the study period and in 10 patients (18.8%), the exacerbations were severe.

Comparisons of characteristics of excluded patients (n = 24) versus those completing the study (n = 53) are shown in Table 2. Plasma IL-6 concentration was significantly higher in the group of excluded patients (n = 24). Sub-group analysis showed no statistical difference in IL-6 values of dropouts (n = 13) versus those completing the study (n = 53). However, patients who died (n = 11) presented with significantly higher values of plasma IL-6 concentration when compared to studied patients (n = 53) [1.5 (1.1 to 2.4) *vs* 0.8 (0.5 to 1.3) pg/ml, p = 0.031]. We found no differences in studied variables between the group of dropouts and patients who died. In addition, the Cox regression including all patients (77) showed that increased IL-6 at baseline was associated with mortality [Hazard Ratio (95% CI) = 2.68 (0.13, 1.84); p = 0.02] after adjustments for age, gender, BODE index, SpO_2_ and comorbidity index.

**Table 2:** Comparison of baseline characteristics of the excluded patients versus those completing the study

**Variables**	**Excluded patients (n = 24)**	**Studied patients (n = 53)**	**p-value**
Gender F/M (n)	8/16	18/35	0.84
Age (years)	65.5 ± 9.3	62.9 ± 8.9	0.23
FEV_1_ (%)	61.3 ± 30.0	56.0 ± 21.1	0.65
FVC (%)	94.7 ± 28.9	88.9 ± 20.9	0.35
FEV_1_/FVC	50.7 ± 13.7	50.5 ± 12.1	0.84
SpO_2_ (%)	92.9 ± 4.9	93.0 ± 3.1	0.49
MMRC (score)	1.6 ± 1.0	1.6 ± 1.0	0.96
6MWD (m)	406.9 ± 105.7	444.0 ± 82.8	0.10
BMI (kg/m^2^)	24.9 ± 5.4	25.6 ± 5.5	0.62
IL-6 (pg/ml)	1.4 ± 1.0	0.9 ± 0.7	**0.04**
CRP (mg/L)	9.7 ± 12.5	8.0 ± 11.8	0.42

Comparisons between excluded patients and those completing the study were performed using the unpaired *t*-test or Mann–Whitney test; p ≤ 0.05. Plus–minus values are presented as mean ± SD. Other values are presented as numbers. *Definition of abbreviations*: Gender F/M = gender female/male; FEV_1_ = forced expiratory volume in the first second (% of predicted); FVC = forced vital capacity (% of predicted); BMI = body mass index; SpO_2_ = pulse oximeter oxygen saturation; MMRC = Modified Medical Research Council; 6MWD = six-minute walking distance; BMI = body mass index; IL-6 = Interleukin 6; CRP = C-reactive protein.

**Figure 2:**
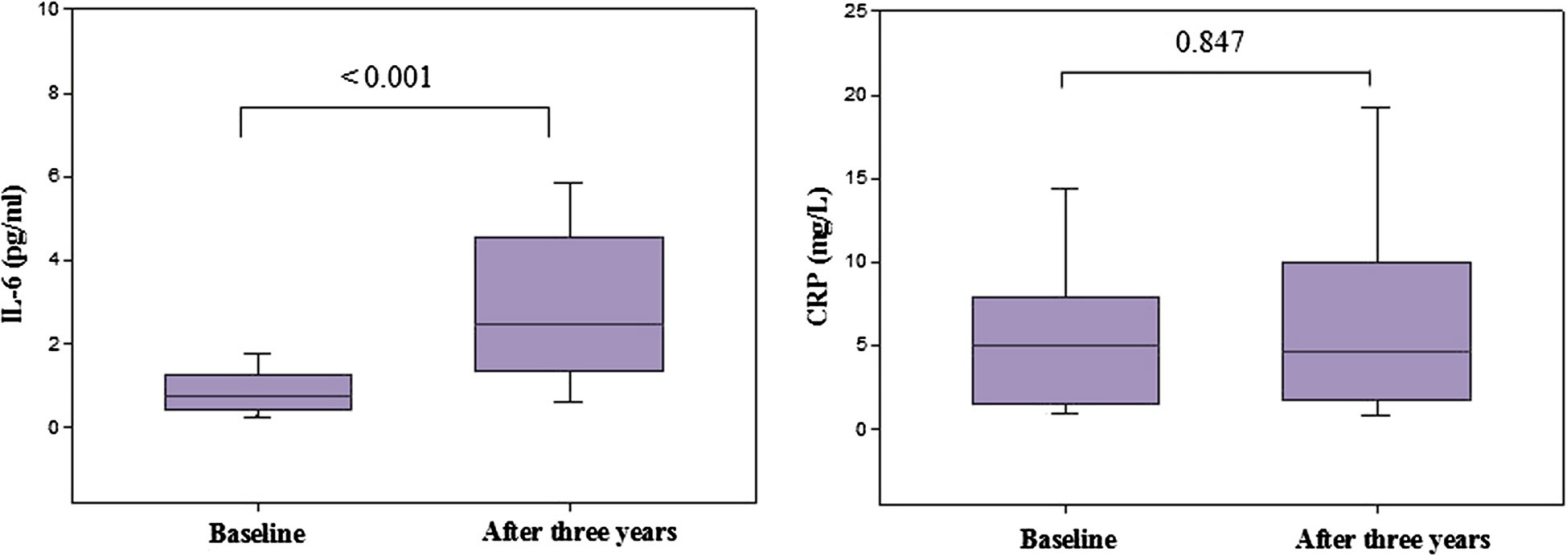
**Plasma concentration of IL-6 and CRP at baseline and after three years.** IL-6 = Interleukin 6; CRP = C-reactive protein. Data show medians with interquartile range.

IL-6 mean values increased significantly after 3 years compared to baseline measurements [0.8 (0.5-1.3) *vs* 2.4 (1.3-4.4) pg/ml; p < 0.001]. Thirty-five patients (66%) had increase > 1 pg/ml of IL-6 over three years. CRP mean values did not change [5 (1.6-7.9) *vs* 4.7 (1.7-10) pg/L; p = 0.84], although eleven patients (21%) presented with changes in CRP >3 mg/L after 3 years (Figure 2). IL-6 was positively correlated with CRP at baseline (r = 0.54; p < 0.001) and after a three-year period (r = 0.53; p < 0.001). No influence of smoking in IL-6 levels was found at baseline and after three years (data not shown).

In the multiple linear regression analysis, male gender was associated with 6MWD values at baseline. Values of IL-6 and FEV_1_, at baseline, were associated with walking capacity at both moments, baseline and after three years (Table 3). There were no associations between inflammatory mediators and BMI and between inflammatory mediators and number of exacerbations. We found no association between CRP and the other studied variables.

**Table 3:** Multiple linear regression models to evaluate the factors associated with baseline and final values of 6MWD

**Variables**	**Baseline 6MWD**	**p-value**	**Final 6MWD**	**p-value**
	**Coefficient (95% CI)**		**Coefficient (95% CI)**	
Gender (Male)	48.36 (8.61, 88.10)	**0.02**	22.64 (−26.55, 71.84)	0.35
Age (years)	−1.40 (−4.77, 1.96)	0.40	−0.60 (−4.88, 3.66)	0.77
FEV_1_ (%)	1.57 (0.74, 2.40)	**<0.001**	1.88 (0.82, 2.95)	**<0.001**
IL-6 (pg/ml)	−42.02 (−65.49, -22.56)	**<0.001**	−54.86 (−82.40, -27.32)	**<0.001**
Charlson index	−8.81 (−33.77, 16.18)	0.48	−11.52 (−43.31, 20.25)	0.46

Baseline (R^2^ = 0.52; p ≤ 0.05) and after three years (R^2^ = 0.44; p ≤ 0.05). *Definition of abbreviations*: 6MWD = six-minute walking distance; FEV_1_ = forced expiratory volume in the first second (% of predicted); IL-6 = Interleukin 6. For both regression analysis the variables values were those obtained at baseline.

## Discussion

To our knowledge, the present study is the first one to show increase over time in systemic inflammation, as measured by plasma IL-6 concentration, in COPD patients. Similar to previous studies [8,10], our results showed that CRP mean values did not change. Furthermore, increased IL-6 levels were associated with mortality and also with reduced exercise tolerance at baseline and after three years. Therefore, our three-year follow-up study showed that IL-6 could be part of the assessment of COPD progression, and increases in serum values are markers of poorer outcomes.

COPD is characterized by a specific pattern of inflammation involving increased numbers of CD8+ T lymphocytes, neutrophils and macrophages in small and large airways and in lung parenchyma and pulmonary vasculature [1]. Alveolar macrophages have a crucial part in orchestrating this inflammation through the release of inflammatory cytokines, such as IL-6, that attract neutrophils into the airways. IL-6 regulates many pathways that could contribute to its effect on inflammatory disease progression. During CD4 T cell differentiation, IL-6 promotes IL-17 and IL-21 production, and suppresses regulatory T cell function. The downstream effect of IL-6 is deposition of matrix, antibody complexes and proteases in the targeted tissue and, consequently, tissue destruction [23]. However, given the cross-sectional nature of most studies, the role of IL-6 over time is not clear. Kolsum et al. [8] showed that the IL-6 mean values did not change significantly during the one-year period, and there was moderate repeatability of IL-6 between the two visits. By contrast, our results reported that IL-6 mean values increased significantly after three years compared to baseline measurements. We also found that patients who died presented higher plasma IL-6 concentration when compared to survivors. Furthermore, increased IL-6 at baseline was associated with mortality after adjustments for age, gender, BODE index, SpO_2_ and comorbidity index. Association between serum IL-6 and prognosis is not conclusive considering the results of recent studies [24,25]. Mehrotra et al. [24] showed that IL-6 was a significant predictor of mortality in 268 elderly subjects with obstructive airway disease. Another study [12] showed association between mortality and levels of WBC counts, IL-6, fibrinogen, CCL-18, CRP, IL-8, and SP-D in 1843 COPD patients studied over three years. Using C statistics, only IL-6 independently added predictive power to the basic clinical model; however, the addition of all the biomarkers in the panel significantly increased the ability of clinical variables to predict mortality in patients with COPD. By contrast, Waschki et al. [25] did not find an association between mortality and levels of IL-6 in 170 outpatients with stable COPD.

CRP mean values did not change over time, although 21% of the patients presented with >3 mg/L changes in CRP after 3 years. We also found no association between CRP levels and mortality or differences in outcome variables between patients who survived at least 3 years with and without increases in CRP >3 mg/L (data not shown). In line with our findings, de Torres et al. [26] showed that baseline serum CRP did not correlate with mortality in patients with moderate to very severe COPD after a three year follow up study. In addition, Pinto-Plata et al. [10] also reported that CRP mean level did not change over a 17-month interval. In contrast, epidemiologic studies showed an association between baseline levels of systemic inflammatory markers and COPD progression [27,28]. In a study with mild to moderate COPD patients, baseline serum levels of CRP were divided into quintiles. After five years of follow-up, it was observed that the highest quintile of CRP was a predictor of mortality compared with the lowest quintile [27]. In the study by Dahl et al. [28], a baseline serum CRP greater than 3 mg/L was associated with increased risk of hospitalization and death after 8 years of follow-up in COPD patients.

As reported in previous studies, we found that CRP levels were positively correlated with plasma IL-6 concentration [4,5,8]; however, while IL-6 levels increased, mean values of CRP did not change over time. Biologically, IL-6 is a primary cytokine regulator of CRP in the liver [7] and may play a salient role in the systemic inflammatory response in COPD [29]. IL-6 has been studied as a COPD marker, since it is increased in low weight COPD patients [9], and its higher levels were associated with lower levels of lung function independent of confounders such as age and smoking [30]. It also plays a critical role in hematopoiesis, causing thrombocytosis and leukocytosis with IL-6 overexpression [31]. In addition, studies have showed that serum IL-6 is a powerful independent predictor of future cardiovascular events in Japanese patients with multiple cardiovascular risk factors [32] and in older adults from a 9-year cohort [33], and the authors suggest that its prognostic value is superior to that of CRP.

We also found that IL-6 was negatively correlated with 6MWD at baseline and after 3 years. Although we cannot prove a causal relation, the association would suggest a persistence deleterious effect of IL-6 on physical performance of COPD patients and further investigation is warranted. In agreement with our results, Brinkley et al. [34] found that IL-6 levels were associated with poorer physical function, independent of age, gender, race, and body composition in older adults across multiple comorbidities, including COPD patients. Yende at al. [35] also showed that IL-6 was an independent predictor of reduced exercise capacity in elderly individuals with an obstructive pattern and with normal spirometry results. In a cross-sectional analysis, Garrod et al. [5] evaluated the values of TNF-α, IL6 and CRP in 41 patients with COPD and found a negative association between CRP and 6MWD, similar to other studies [4,10]. These results provide evidence that chronic inflammation and impaired physical function are related in various age-related diseases. Our study, in line with this hypothesis, shows that inflammation is persistent and associated with exercise tolerance over time in COPD patients.

We also explored the relationship between number of exacerbations and plasma IL-6 and CRP concentration; however, no association was found. Agusti et al. [11] showed that the annual rate of exacerbations during the 3 year follow-up were higher in the persistently inflamed patients, compared with non-inflamed patients. However, the logistic regression did not show association between that annual rate of exacerbations and the presence of persistent systemic inflammation (defined as in upper quartile at baseline and after one-year for at least 2 biomarkers).

The strengths of this study are the wide range of disease severities designed to evaluate changes in systemic inflammation and its relationship to disease prognosis outcomes over a considerable period of time. Despite of the small number of patients, this study shows the association of IL-6 with prognosis in COPD patients and reinforces the role of this mediator in the evaluation of patients’ outcomes. In addition, taking in consideration the difference in values of serum IL-6 between healthy subjects and smokers in previous study [3], a sample of 40 COPD patients would be necessary (α = 0.05; power = 80%). However, a large sample size could allow the identification of a reliable cutoff value of IL-6 to predict poorer prognosis. We measured the systemic inflammatory mediators at baseline and after three years; therefore our study has limitations regarding the predictor value of serum IL-6 for shorter periods of time.

## Conclusions

The systemic inflammatory process, evaluated by IL-6, seems to be persistent and progressive in COPD patients. In addition, increased systemic inflammation is associated with mortality and with reduced exercise tolerance over time. These results suggest that inflammatory markers could contribute to evaluate outcomes related to COPD progression.

## Abbreviations

6MWD: Six-minute walk distance; BMI: Body mass index; COPD: Chronic obstructive pulmonary disease; CRP: C-reactive protein; BTS: Brazilian Thoracic Society; FEV_1_: Forced expiratory volume in 1 second; FVC: Forced expiratory vital capacity; GOLD: Global initiative for chronic obstructive lung disease; IL-6: Interleukin 6; MMRC: Modified Medical Research Council; SpO_2_: Pulse oximeter oxygen saturation.

## Competing interests

The authors declare that they have no competing interests.

## Authors’ contributions

RF and IG conceptualized the study. SET and RF performed statistical analysis; RF, SET and IG analyzed the data and drafted the manuscript. RF, LMOC, CC and CC contributed to cohort enrollment/data collection. All authors contributed to the writing of this manuscript, and they read and approved of the final draft of the manuscript.

## Supplementary Material

Additional file 1 Three-year follow-up of Interleukin 6 and C-reactive protein in chronic obstructive pulmonary disease. Click here for file
