# Supplementary material for: Three-year follow-up of Interleukin 6 and C-reactive protein in chronic obstructive pulmonary disease
Source: Respir Res. 2013 Feb 20;14(1):24. doi: 10.1186/1465-9921-14-24 (PMC3620569; doi:10.1186/1465-9921-14-24)
Supplement: Additional file 1 — Three-year follow-up of Interleukin 6 and C-reactive protein in chronic obstructive pulmonary disease. [file 1465-9921-14-24-S1.doc]

**Additional file**

**Three-year follow-up of Interleukin 6 and C-reactive protein in chronic obstructive pulmonary disease**

Renata Ferrari, Suzana E Tanni, Laura M O Caram, Corina Corrêa, Camila R Corrêa, Irma Godoy

**Protocol**

Patients’ medical therapy and disease severity were categorized according to GOLD and BTS guidelines [1,2]. Patients with chronic hypoxemia received a stable dose of oxygen therapy over the 6 months prior to study enrollment.

Patients were evaluated at baseline and attended the clinics every 6 months for 3 years or until death. Patients or family, in the case of death, were interviewed by telephone every 3 months to identify data associated with exacerbation and/or hospitalizations. Data were confirmed during visits at the clinics and by reviewing medical records. Basic causes of death were reviewed on death certificates. After 3 years, the measurements were repeated in clinically stable patients.

***Exacerbations****:* Information on exacerbations was not available at baseline and was collected during the study period. During the telephone interview a structured questionnaire was used to identify data associated with exacerbation and/or hospitalizations (Questionnaire 1). An exacerbation was defined as moderate (requiring a visit to a doctor or the emergency department and treatment with antibiotics or systemic steroids or both) or severe type II (requiring hospital admission) [3]. Mild exacerbations not requiring intervention were not included in the study.

***Exercise tolerance:*** The six-minute walk distance (6MWD) was performed according to the guidelines of the American Thoracic Society [4]. Patients were instructed to walk, attempting to cover as much ground as possible within 6 min. A research assistant timed the walk, and standardized verbal encouragement was given. Following a rest of at least 30 min, each subject performed a second 6MWD in the same manner as the first. Pulse oximeter oxygen saturation (SpO_2_) was monitored throughout the test. Patients who were hypoxic at baseline and patients whose SpO_2_ decreased to <85% during the test were given oxygen by a physical therapist who wheeled an oxygen tank in a handcart alongside the patient. Before and after the test, data were obtained for SpO_2_, heart rate, respiratory rate, dyspnea sensation (Borg scale dyspnea score) and blood pressure. The distance covered is reported in meters.

1. Global Initiative for Chronic Obstructive Lung Disease guideline: global strategy for the diagnosis, management and prevention of chronic obstructive pulmonary disease (updated 2010). Global Initiative for Chronic Obstructive Lung Disease website.<http://www.goldcopd.com/>. Accessed June 15 2012.

2. Jardim JR, Oliveira JA, Nascimento O: **II Consenso Brasileiro de DPOC.** *J Bras Pneumol* 2004, **30**(Suppl 5):1-42.

3. Rodriguez-Roisin R: **Toward a consensus definition for COPD exacerbations.** *Chest* 2000, **117**(Suppl 2)**:**398-401.

4. American Thoracic Society statement: **Guidelines for the six-minute walk test.** *Am J Respir Crit Care Med* 2002, **166:**111-117.

Questionnaire 1

Exacerbation: presence of two or more symptoms and at least one change in the maintenance treatment

( ) yes ( ) no

- Symptoms:

( ) Increased dyspnea

( ) Increased volume of sputum

( ) Increased purulence of sputum

( ) Increased cough

- Treatment (requiring a visit to a doctor or the emergency department):

( ) treatment with antibiotics

( ) systemic steroids

Hospitalization: ( ) yes ( ) no

- Cause of hospitalization: *____________________________________*

Death*:* ( ) yes ( ) no

- Cause of death:*_____________________________________________*
